# Prognostic Frailty-Based Determinants of Long-Term Mortality in Older Patients with Newly Diagnosed Multiple Myeloma

**DOI:** 10.3390/cancers17050789

**Published:** 2025-02-25

**Authors:** Mariya Muzyka, Silvia Ottaviani, Irene Caffa, Tommaso Bonfiglio, Erica Parisi, Ana Guijarro, Luca Tagliafico, Roberto Massimo Lemoli, Marta Ponzano, Cristina Marelli, Alessio Signori, Alessio Nencioni, Michele Cea, Fiammetta Monacelli

**Affiliations:** 1 Department of Internal Medicine and Medical Specialties, University of Genoa, 16132 Genoa, Italy; 2 Ospedale Policlinico San Martino IRCCS, 16132 Genoa, Italy; 3 Department of Health Sciences, Section of Biostatistics, University of Genoa, 16132 Genoa, Italy

**Keywords:** multiple myeloma, prognosis, geriatric assessment, frailty, survival, hospitalization

## Abstract

**Background/Objectives:** Multiple myeloma (MM) is a plasma cell neoplasm predominantly diagnosed in older adults. However, the significance of defining patient frailty, as well as identifying the most suitable and reliable tools for its assessment, remains to be firmly established. **Methods**: This retrospective observational study investigated 36 patients aged 65 or older who underwent Comprehensive Geriatric Assessment (CGA). The average patient age was 76 (SD 6.22), with 33.3% being female. Patients were evaluated using the International Myeloma Working Group Frailty Index (IMWG-FI) and the 40-item Rockwood’s Frailty Index (FI) at the Oncogeriatrics clinic of the IRCCS Polyclinic San Martino Hospital, Genoa, Italy between December 2017 and August 2021. Laboratory, cancer-specific, demographic, and clinical variables were collected. Survival analysis and frailty comparison were conducted using Stata version 17.0. **Results**: Stepwise multivariate analysis identified the Numerical Rating Scale (NRS) (HR 1.40, 95% CI 1.09–1.78, *p* = 0.008) and Rockwood’s Frailty Index (FI) (HR 2.23, 95% CI 1.29–3.87, *p* = 0.004) as significant prognostic predictors, adjusted for sex, ISS stage, and multimorbility. Comparison between Rockwood’s FI and IMWG-FI using Spearman correlation coefficient showed no statistically significant correlation (r = 0.268, *p* = 0.114). Multivariate Cox model, adjusting for sex, International Staging System (ISS) stage, and Cumulative Illness Rating Scale (CIRS) comorbidity index demonstrated the superior predictive ability of Rockwood’s FI over IMWG-FI (C-index 0.775 vs. 0.749). **Conclusions**: The 40-item Rockwood FI emerges as a valuable tool for prognostication in old MM patients, demonstrating non-inferiority to the traditional IMWG-FI in predictive accuracy, emphasizing the importance of a comprehensive approach considering both disease-specific and patient-related factors.

## Simple Summary

This study analyzed 36 older adults (≥65 years, average age 76, 33.3% female) with multiple myeloma (MM) who underwent comprehensive geriatric assessment at IRCCS Polyclinic San Martino Hospital, Genoa between 2017 and 2021. Patients were evaluated using the International Myeloma Working Group Frailty Index (IMWG-FI) and the 40-item Rockwood Frailty Index (FI). Multivariate analysis identified Rockwood’s FI as a key prognostic factor, outperforming IMWG-FI (C-index 0.775 vs. 0.749). Spearman correlation showed no significant relationship between the two indices (r = 0.268, *p* = 0.114). These findings highlight the superior predictive value of Rockwood FI and emphasize the need for comprehensive, patient-centered assessments in older adults with MM.

## 1. Introduction

Multiple myeloma (MM) is a plasma cells tumor that typically occurs in older patients: the median age at the time of diagnosis is 69 years, and more than 30% of these are older than 75 years [1]. As a result, given the global increase in the elderly population, the incidence of MM has constantly increased in recent years [2,3]. Importantly, old-age patients represent a highly heterogeneous population with different proportions of multimorbid and frail adults, who are more vulnerable to adverse clinical outcomes; such features often raise uncertainties about the clinical benefit derived from cancer treatments in these patients [4]. In such a context, although clinical benefits in terms of increased progression-free survival (PFS) and overall survival (OS) are derived from novel therapies (such as immunomodulators, proteasome inhibitors, and monoclonal antibodies) and supportive care availability, chronological age is still often recognized as a major determinant of outcomes rather than biological age [5,6,7,8].

As a result, great caution should be used in the management of old-age patients, with several clinical scores developed for the identification of frail and unfit subjects. However, chronological age and performance status alone are insufficient to classify patients’ frailty [9,10,11] which identifies a geriatric syndrome characterized by impaired biological and functional reserves that reduce the ability to respond to stressors [12]. To date, although two key relevant frailty constructs have been developed—such as Linda Fried’s physical frailty phenotype [13] and Kenneth Rockwood’s deficit accumulation model [14], both of which address the clinical complexity of older adults—frailty assessment in clinical practice still remains challenging due to heterogeneity and complexity of its measurement. At diagnosis, at least 1/3 of MM patients have some degree of frailty [15], and growing evidence suggests that it is associated with reduced therapeutic response, increased toxicity, worse survival [16], and a higher risk of treatment discontinuation [17].

The International Myeloma Working Group Frailty Index (IMWG-FI) represents the currently most used system to evaluate the fitness of MM patients and define them as “fit”, “intermediate fit”, or “fragile”; a significant impact of this evaluation on both PFS and OS has been demonstrated [18]. However, Scheepers et al. have recently shown the accuracy and appropriateness of a Comprehensive Geriatric Assessment (CGA) for the identification of frailty in older adults with hematologic malignancies: 68% (range 25–76%) of patients according to CGA assessment resulted in being frail compared to 45% as defined by other approaches [16]. Since older patients with geriatric disabilities have significant clinical benefits, in terms of enhanced responses and reduced adverse events occurrence, from a timely frailty status assessment, the CGA represents the most accurate system to define fitness and thus for selecting optimal strategies for old age patients [19,20].

In line with these data, Rockwood’s Frailty Index (FI) is considered the gold standard and the most widely used tool for frailty assessment and has been widely validated across various populations; for instance, it has been applied to patients with myelodysplastic syndromes, demonstrating that age-adjusted IPSS-R score, FI, and Charlson Comorbidity Index were independent prognostic determinants for overall survival. Indeed, frailty and comorbidity scores resulted in improved IPSS-R risk stratification by 30% and 5%, respectively [21]. Conversely, Mian et al., applied the frailty index paradigm to a cohort of MM patients and observed that each 10% increase in frailty index was associated with a 16% increase in the risk of death. The estimated median overall survival of frail patients was 26.8 months, compared to 43.7 months for those who were not frail [22]. Similarly, in a cohort of 3807 U.S. veterans aged 65 or older Patel et al. observed that a higher FI score was associated with higher mortality with hazard ratios of 1.33, 1.97, 2.86, and 3.22 for prefrail, mildly frail, moderately frail, and severely frail individuals, respectively [23].

Given this background, we retrospectively compared the prognostic accuracy of IMWG-FI and the Italian translation of the 40-item Rockwood FI [24] to predict overall survival (OS) in a cohort of newly diagnosed non-transplant eligible MM patients.

## 2. Materials and Methods

### 2.1. Patient Population and Study Designs

This retrospective observational study included 36 patients with non-transplant-eligible MM referred to the oncogeriatrics clinic of the IRCCS Polyclinic San Martino Hospital, Genoa, Italy between December 2017 and August 2021. Inclusion criteria were patients aged 65 or older newly diagnosed with MM not eligible for high-dose therapies who underwent a CGA at diagnosis and received treatment at the oncogeriatrics clinic during the specified period with complete clinical data available. Patients were classified as NTE if they were unable to safely undergo autologous stem cell transplantation (ASCT) due to advanced age, significant comorbidities (e.g., cardiovascular conditions such as heart failure or recent myocardial infarction), or poor functional status (e.g., ECOG performance status ≥ 2 or a Karnofsky Performance Score < 60%).

Exclusion criteria included age below 65 years, patients with new diagnosis of MM transplant eligible, monoclonal gammopathy of undetermined significance (MGUS) or smoldering myeloma, absence of CGA or complete clinical data, and treatment received outside the specific clinic.

### 2.2. Laboratory and Cancer-Specific Variables

Disease-related variables included SLiM-CRAB criteria present at diagnosis [20], histotype, cytogenetic risk status assessed by fluorescence in situ hybridization (FISH), bone disease, extramedullary disease, flow cytometric data from bone marrow aspirates (BM), and disease staging system according to Durie–Salmon (D-S) [25] and to the International Staging System (ISS) [26], if available. Laboratory variables included blood count, creatinine, urea, coagulation profile (international normalized ratio, prothrombin time, partial thromboplastin time), calcium, serum albumin, total serum proteins, total and direct bilirubin, alanine transaminase, aspartate transaminase, beta2-microglobulin, lactate dehydrogenase, serum free light chain ratio, M component, 24-h proteinuria, and 24 h Bence Jones proteinuria.

### 2.3. Demographic and Clinical Assessments

Demographic data included age and sex. Following an initial frailty screening using the G8 tool [27] (with, a cut-off of ≤14), each patient underwent a CGA at MM diagnosis, including cognitive status using Mini-Mental State Examination (MMSE) and Clock Drawing test according to Shulman (CDT) [28,29]. Nutritional risk was assessed using Mini Nutritional Assessment (MNA) [30]; functional autonomy with Barthel Index and instrumental activity of daily living (IADL) [31,32]: multimorbidity with the Cumulative Illness Rating Scale (CIRS) [33]; psycho-affective status with 15-item Geriatric Depression Scale (GDS) [34]; gait and risk of falling with the Tinetti Scale [35], and pain evaluation with the Numerical Rating Scale (NRS) [36]. Social vulnerability was assessed with the Gijon scale [37] and sarcopenia and physical performance with a hand grip (HG) test (using a GIMA 28,791 Smedley dynamometer), SARC-F scale [38] and the Timed-Up and Go test (TUG) [39,40]. Polypharmacy was assessed by counting the number of drugs [41] and quality of life was assessed with the EuroQol instrument (EQ-5D) [42]. A CGA cut-off>3 defined frailty [43].

For frailty stratification, the IMWG-FI and the Rockwood’s FI were calculated. IMWG score includes the patient’s age and CGA measurements including Activities of Daily Living (ADL), instrumental activity of daily living (IADL), and comorbidity (Charlson Comorbidity Index (CCI) [44]). The IMWG scoring system indicates frailty levels: fit (score = 0), intermediate-fit (score = 1), and frail (score ≥ 2).

Rockwood’s FI considers 40 health deficits, with cumulative scores indicating frailty levels: fit (score ≤ 0.08); prefrail (score: 0.09 < pre frail < 0.24); frail (score ≥ 0.25). See File S1 for tool details.

Time to death from all causes between the first geriatric visit at MM diagnosis and death was recorded. The date of censorship was 31 August 2022.

### 2.4. Statistical Analysis

Results were described using mean and standard deviation (SD) or median and interquartile range (IQR) or absolute frequency (N) and relative frequency (%) according to the distribution. The normal distribution hypothesis was checked through the Shapiro–Wilk test. The Spearman correlation coefficient assessed the correlation between IMWG-FI and Rockwood’s FI. Characteristics of alive or deceased patients were compared through the *t*-test, the Wilcoxon sum of ranks test, or the chi-squared test, according to the distribution. The Kaplan–Meier survival curve was used to assess time to death and univariate Cox proportional hazards models were performed to evaluate the impact of the variables on mortality. A multivariate Cox model with backward stepwise selection was run including variables with a *p*-value < 0.05 in the univariate analysis and additionally including sex, ISS stage, and CIRS and comorbidity index due to clinical relevance. Moreover, variables with higher degree of collinearity were excluded [45].

A multivariate model including all the selected variables with backward stepwise selection and adjusting for age was performed for sensitivity analysis. The final stepwise multivariate model was adjusted for age. The proportional hazard assumption of the final multivariate model was checked using Schoenfeld residuals. Rockwood’s FI and IMWG-FI were compared using the C-index, the AIC and BIC values, adjusting for sex, ISS stage, and CIRS comorbidity index and the same analysis was performed to assess sensitivity. DeLong’s test was used to assess the statistical significance of the difference in C-index between the models.

All tests were two-sided, and *p*-values < 0.05 were considered statistically significant. All analyses used Stata version 17.0 (Stata Corporation, College Station, TX, USA).

## 3. Results

### 3.1. Cohort Characteristics

Between December 2017 and August 2021, 54 consecutive newly diagnosed MM patients who were non-transplant-eligible were screened. Of 54 admitted patients, 36 were included in the final analysis. Of these, 4 patients were excluded for a diagnosis of MGUS, and 1 patient was excluded for a diagnosis of smoldering MM; a further 13 patients were excluded because CGA assessment was performed more than 60 days after the diagnosis of MM. On average, CGA was performed 17 days after diagnosis (range: 2–57 days) and always before treatment initiation. Baseline characteristics detailed in Table 1, showed a mean age of 76 (±6.22) years (range: 65–92 years) with 33% aged over 80 and 67% male. Most patients had IgG histotype at diagnosis (64%); the most-represented disease stages were IIA and IIIA according to Durie–Salmon and II and III based on the ISS system; a minor prevalence was observed for Stage I. A standard cytogenetic risk was observed in the majority of included patients (80%) with at least one bone lesion observed at the time of diagnosis in almost all cases. Extramedullary disease was observed in only 2 patients. Detailed blood and urine tests and cytofluorimetric data are illustrated in File S2.

### 3.2. CGA and Frailty Assessment

On the basis of CGA, almost half of the patients (44%) were at higher malnutrition risk, 39% had impaired instrumental activities and had a mean of 4 comorbidities and 5 drugs, respectively.

We subsequently compared the fitness of included patient by measuring their frailty scores derived from the IMWG-FI and Rockwood’s FI. As shown in Table 1, the IMWG-FI mean score was 1 (IQR 0–2), and the Rockwood FI mean score was 0.18 (IQR 0.11–0.23). These data suggest an imbalance between these systems: indeed, 64% of patients resulted in pre-frail based on Rockwood’s FI, while most patients were classified as frail according to IMWG-FI.

### 3.3. Outcomes

**Figure 1:**
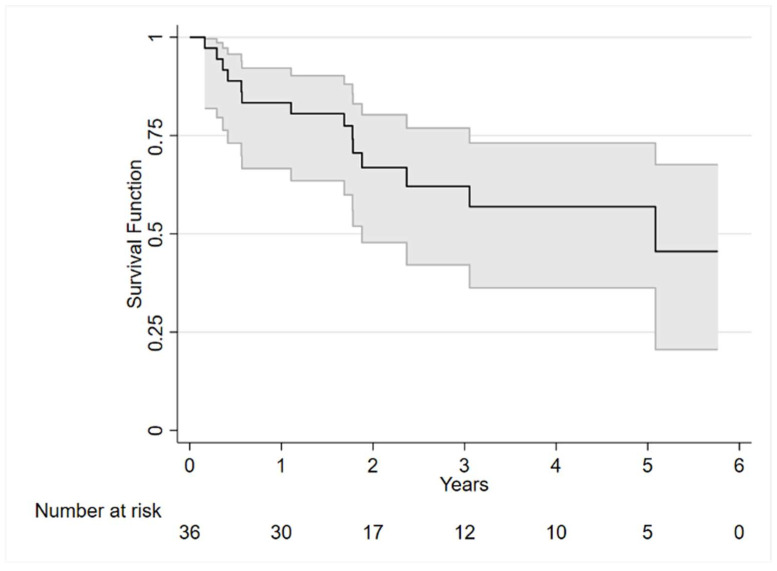
Kaplan–Meier graph estimating the survival probability over time.

According to the censorship data, 14 patients (39%) died, with 14% passing away within the first year. Specifically, 3 patients died of disease progression and related complications, 2 of COVID-19 pneumonia, 1 of acute pulmonary edema, and the cause of death for the remaining 8 patients was unknown. Figure 1 illustrates the Kaplan–Meier curve, depicting the mortality trend within the cohort over 5.77 years. As detailed in Table 1, surviving patients demonstrated higher levels of functional autonomy in instrumental activities (*p* = 0.017), and lower disease severity according to the Durie–Salmon score (*p* = 0.048).

First-line treatments in the study cohort included velcade melphalan prednisone (VMP), lenalidomide dexamethasone (RD), daratumumab lenalidomide dexamethasone (DRD), and velcade lenalidomide dexamethasone (VRD). Treatment responses varied, with 14 patients experiencing progression disease (PD), while 22 achieved some level of clinical benefit, including partial response (PR, N = 5), stable disease (SD, N = 5), very good partial response (VGPR, N = 10), and complete response (CR, N = 2).

Following a preliminary univariate survival analysis, the stepwise multivariate analysis identified NRS (HR 1.40, 95% CI 1.09–1.78, *p* = 0.008) and Rockwood’s FI (HR 2.23, 95% CI 1.29–3.87, *p* = 0.004) as significant predictors, along with age (HR 0.15, 95% CI 0.03–0.75, *p* = 0.021) and advanced stage of disease (HR 6.09, 95% CI 1.36–27.21, *p* = 0.018). Sex, ISS stage, and CIRS comorbidity index were included in the model as adjustments (Table 2). File S3 contains univariable Cox proportional hazards model for death for laboratory and MM-specific variables.

To compare the predictive ability of the models between Rockwood’s FI and IMWG-FI, we first evaluated the correlation between IMWG-FI and Rockwood’s FI using the Spearman correlation coefficient, which was found not to be statistically significant: r = 0.268 (*p* = 0.114). Subsequently, a multivariate Cox model was built with the variable of interest (IMWG-FI vs. Rockwood’s FI) and adjustment for sex, ISS stage, and CIRS comorbidity index. AIC, BIC, and C-index were then calculated for comparison. The data presented in Table 3 indicate that the model including Rockwood’s FI showed superior predictive ability regarding the survival of older patients with MM, with equal stage of disease and multimorbidity burden (C-index 0.775 vs. 0.749). Sensitivity analysis, adjusted for age, yielded consistent results with those reported above. However, the *p*-value from DeLong’s test was 0.505, indicating that the difference in C-index between the models was not statistically significant.

## 4. Discussion

MM is predominantly a disease of old-age adults. Thus, geriatric assessment is emerging as crucial to identifying the vulnerabilities and frailties that compromise treatment outcomes and susceptibility to adverse events derived from different therapies. Older adults with MM represent a growing demographic, warranting personalized treatments based on biological health status to improve survival, maintenance of functional reserve, and quality of life. While existing evidence has focused on cancer-related prognostic variables and validated myeloma-specific scores to delineate fitness versus frailty status in older adults, the full understanding of the interplay between frailty and cancer survival remains incomplete, as does the systematic application of such scores in routine practice. In assessing older adults with MM, both cancer-specific and patient-specific factors may undermine functional reserve, influencing prognosis, complicating treatment decisions, toxicity assessment, and shared decision-making processes.

This study paves the way to integrate laboratory and MM-specific variables with patients’ frailty, employing a methodologically robust stratification to predict OS in a real-world cohort of older newly diagnosed MM patients non-eligible for HD-therapies. Our findings demonstrated that the 40-item Rockwood FI was significantly associated with OS, demonstrating non-inferiority to the gold standard IMWG-FI along with a comprehensive set of MM-specific variables. These original findings underscore how patient-based frailty stratification impacts MM clinical management, progression, and outcomes.

Notably, the study population was aged over 75 years, providing valuable insights into frailty measurement applicability in very old individuals.

Currently available clinical scores, including IMWG-FI, classify patients as “fit”, “intermediate-fit” or “frail” based on chronological age, with patients over 80 automatically being classified as frail [46]. However, with the increasing clinical heterogeneity of aging populations, relying solely on chronological age risks undertreating patients who may benefit from more intensive therapeutic regimens. In such a context, our frailty stratification identified vulnerable patients as at intermediate risk, including one-third of the oldest old. Notably, our study revealed that chronological age alone did not correlate with an increased likelihood of frailty.

Moreover, by incorporating a series of disease-related prognostic factors into the predictive model, our approach captures the clinical complexity of MM and strengthens the prognostic ability of Rockwood’s FI. Unlike the IMWG-FI, the Rockwood FI, as part of a CGA, not only serves as a prognostic tool but also offers valuable insights into patients’ functional and clinical vulnerabilities. This enables the identification of specific targets for tailored interventions, reinforcing the role of multidimensional frailty assessment in optimizing the management of older MM patients beyond mere risk stratification.

Among the evidence in the literature, Stege et al. demonstrated that older patients aged 75 years and older with newly diagnosed MM categorized as frail based on IMWG-FI scoring had comparable outcomes to patients classified as intermediate fit [43]. Moreover, Murillo et al. underscored that frailty-based stratification with Fried’s physical phenotype outperformed IMWG-FI classification, suggesting that biological age rather than chronological age plays a critical role in informing outcomes [47].

Mian et al. utilized Rockwood’s deficit accumulation model on a large sample of older patients without cancer to derive a frailty index applicable to MM patients. This final 25-item model was associated with OS even after adjusting for chronological age, emphasizing the differences between biological and chronological age [20]. Particularly, in the cohort of newly diagnosed MM, the mean frailty index was 0.28, and each 10% increase in frailty index was associated with a 16% increased risk of death, with a median OS estimated at 26.8 months. In our study, a 0.1 increase in Rockwood’s FI resulted in a 112% increase in the risk of death at the end of the observation period.

Our findings contrast with those reported by Tyczynska et al., who concluded that the management of patients over 75 was not dependent on frailty assessment [48]. However, in that cohort, most patients had a relatively good performance status, potentially undermining the generalizability of the findings due to the lack of robust frailty stratification.

It could be hypothesized that the cumulative burden of clinical deficits, including comorbidity, functional impairment, mental status, general health status, and quality of life, may be related to the overall burden of MM, ultimately increasing the likelihood of mortality. Rockwood’s FI captures the dynamic changes of frailty when superimposed with major stressors, such as MM and cancer therapies, influencing functional reserve; remission; disease-free progression; and outcomes, such as long-term mortality; disability; and quality of life.

Our study has important limitations, including the small sample size, the single-centre enrolment and its retrospective nature. The study sample is skewed toward males but is consistent with data from the literature [49]. Additionally, the small number of patients and “death” events limited our ability to establish the prognostic value of the 40-item Rockwood FI. Furthermore, other important outcomes such as PFS, therapeutic regimen, or hospitalization were not reported, warranting further in-depth analysis.

Despite these limitations, the study offers a comprehensive evaluation of old-age MM patients, adopting a methodologically robust frailty assessment. Focusing on older adults aged over 75 years, this study addresses the unique needs and challenges faced by this growing demographic within the MM patient population. Our findings, though preliminary, have real-world applicability, providing actionable insights that can inform and enhance clinical practice.

The Rockwood FI enables a more precise stratification of frailty in older patients, guiding therapy decisions to balance efficacy and safety. It assists in selecting treatment intensity, adjusting doses based on functional reserves, and assessing the risk of treatment-related toxicities. Additionally, its dynamic nature allows for ongoing frailty assessments, enabling treatment adjustments as the clinical status evolves. Future research will prospectively evaluate frailty trajectories and the impact of targeted geriatric interventions on key clinical outcomes, including survival, quality of life, treatment discontinuation, and dose reduction rates. In such a complex therapeutic landscape, with the availability of increasingly effective treatments, such as bispecific antibodies and CAR-T strategies, the in-depth analysis of MM patient’s frailty is becoming imperative.

## 5. Conclusions

In conclusion, the 40-item Rockwood FI frailty stratification model, by demonstrating effectiveness in predicting time to death in older patients with MM, establishes itself as a valuable and innovative prognostic tool. Our study underscores the importance of precise patient selection, highlighting the limitations of relying solely on chronological age for patient phenotyping.

While Rockwood’s FI effectively captures the dynamic nature of frailty and its interplay with the disease burden of MM, the limited sample size of this study necessitates caution in interpreting these findings. Prospective validation in larger cohorts will be essential to confirm its utility and to explore how frailty assessments can further enhance prognostic accuracy and guide personalized treatment strategies in clinical practice.

## Figures and Tables

**Table 1:** Baseline characteristics of the population.

	Overall, N = 36 (100%)	Dead, N = 14 (39%)	Alive, N = 22 (61%)	*p*-Value
Female sex	12 (33.33%)	3 (21.43%)	9 (40.91%)	0.227
Age	76 (6.22)	78.21 (6.76)	74.59 (5.56)	0.089
IMWG-FI, median (IQR)	1 [0–2]	1.5 [1–3]	1 [0–2]	0.367
G8 (≤14), median (IQR)	N = 35 13 [12–15]	N = 13 12 [11–14]	N = 22 13.25 [12–16]	0.130
MMSE, median (IQR)	28 [26.6–29]	27.85 [26.4–28.7]	28 [27–29]	0.442
Clock drawing test, median (IQR)	N = 35 2 [1–2]	N = 14 2 [1–3]	N = 21 1 [1–2]	0.137
MNA	23.93 (3.68)	22.54 (3.37)	24.82 (3.66)	0.069
IADL, median (IQR)	7 [7–8]	8 [7–8]	8 [8–8]	0.017
Barthel, median (IQR)	100 [95–100]	97.5 [90–100]	100 [95–100]	0.381
CIRS comorbidity index	4.29 (1.92)	4.74 (2.11)	4 (1.77)	0.263
GDS, median (IQR)	N = 35 3 [1–4]	N = 14 3.5 [1–6]	N = 21 3 [2–3]	0.388
Tinetti test, median (IQR)	N = 34 26 [22–28]	N = 13 23 [21–27]	N = 21 26 [2–28]	0.276
NRS, median (IQR)	4 [0–5]	5 [3–6]	2 [0–5]	0.057
Gjion	8.75 (2.66)	8.57 (2.38)	8.86 (2.87)	0.753
TUG, median (IQR)	N = 34 9.5 [7.2–12]	N = 13 10 [7.2–12]	N = 21 8.5 [8–11]	0.644
HG	27.92 (9.16)	29.76 (8.32)	26.75 (9.67)	0.345
SARC-F	N = 20 2.85 (2.39)	N = 8 3.13 (2.42)	N = 12 2.67 (2.46)	0.686
Cut-off CGA (≥3)	3.19 (2.15)	3.71 (2.16)	2.86 (2.12)	0.253
Polypharmacy	6.22 (3.45)	6.93 (3.79)	5.77 (3.22)	0.334
Rockwood’s FI, median (IQR)	0.18 [0.11–0.23]	0.21 [0.18–0.27]	0.16 [0.1–0.2]	0.066
EuroQoL	N = 35 0.70 (0.17)	N = 13 0.63 (0.20)	N = 22 0.74 (0.14)	0.072
RT, yes	N = 32 10 (31.25%)	N = 14 4 (28.57%)	N = 18 6 (33.33%)	0.773
Stage D-S	N = 35	N = 14	N = 21	0.048
IA	5 (14.29%)	0 (0.00%)	5 (23.81%)	
IIA	11 (31.43%)	6 (42.86%)	5 (23.81%)	
IIIA	12 (34.29%)	7 (50.00%)	5 (23.81%)	
IIIB	7 (20.00%)	1 (7.14%)	6 (28.57%)	
ISS stage				0.221
I	8 (22.22%)	1 (7.14%)	7 (31.82%)	
II	13 (36.11%)	6 (42.86%)	7 (31.82%)	
III	15 (41.67%)	7 (50.00%)	8 (36.36%)	

(i) The entire population; (ii) dead patients; (iii) living patients. Results are presented through mean and standard deviation (SD) and absolute frequency (N) and relative frequency (%), for continuous and categorical variables respectively, unless otherwise specified. *p*-values of the *t*-test, the Wilcoxon sum of ranks test, or the chi-squared test are presented to compare (ii) and (iii) according to the distribution. Abbreviation list. MMSE: Mini-Mental State Examination; MNA: Mini Nutritional Assessment; IADL: instrumental activity of daily living; CIRS: Cumulative Illness Rating Scale; GDS: 15-item Geriatric Depression Scale; NRS: Numerical Rating Scale; HG: hand grip; TUG: Timed Up and Go test; Rockwood’s FI: Rockwood’s Frailty Index; RT: radiotherapy; D-S: Durie–Salmon; ISS: International Staging System. HR: hazard ratio; CI: confidence interval; IQR: interquartile range.

**Table 2:** Univariate and backward stepwise multivariate Cox proportional hazards model for death.

	Univariate		Backward Stepwise Multivariate ^#^	
	HR (95% CI)	*p*-Value	HR (95% CI)	*p*-Value
Sex (female vs. male)	0.55 (0.15–1.96)	0.354	0.15 (0.03–0.75)	0.021
Age	1.10 (1.01–1.19)	0.022 *	-	-
IMWG-FI	1.67 (1.09–2.57)	0.019	-	-
G8 (≤14) (N = 35)	0.76 (0.60–0.95)	0.016	-	-
MMSE	0.90 (0.70–1.14)	0.368	-	-
Clock drawing test (N = 35)	1.43 (0.90–2.27)	0.127	-	-
MNA	0.81 (0.69–0.95)	0.008 *	-	-
IADL	0.73 (0.56–0.96)	0.024 *	-	-
Barthel index	0.96 (0.91–1.01)	0.158	-	-
CIRS comorbidity index	1.21 (0.94–1.54)	0.137	-	-
GDS (N = 35)	1.27 (1.04–1.55)	0.021	-	-
Tinetti (N = 34)	0.92 (0.81–1.04)	0.195	-	-
NRS	1.25 (1.02–1.54)	0.034	1.40 (1.09–1.78)	0.008
Gjion	1.05 (0.86–1.29)	0.626	-	-
TUG (N = 34)	1.07 (0.94–1.22)	0.311	-	-
HG	1.01 (0.96–1.06)	0.781	-	-
SARC F (N = 20)	1.10 (0.80–1.51)	0.556	-	-
Cut-off CGA (≥3)	1.36 (1.03–1.79)	0.027 *	-	-
Polypharmacy	1.12 (0.96–1.31)	0.139	-	-
Rockwood’s FI (0.1 increase)	1.57 (1.07–2.31)	0.021	2.23 (1.29–3.87)	0.004
EuroQoL (0.1 increase) (N = 35)	0.75 (0.55–1.02)	0.066	-	-
RT (yes vs. no) (N = 32)	0.68 (0.21–2.22)	0.525	-	-
Stage D-S (IA or IIA vs. IIIA or IIIB) (N = 35)	0.59 (0.20–1.76)	0.348	-	-
ISS stage (I or II vs. III)	0.36 (0.12–1.10)	0.072	6.09 (1.36–27.21)	0.018

HR: hazard ratio; CI: confidence interval. ^#^ Multivariate Cox model with backward stepwise selection, including sex, ISS stage, and CIRS and comorbidity index due to clinical relevance. * Excluded from the multivariate model due to the high correlation with another variable included.

**Table 3:** AIC, BIC, and C-index for the comparison between Rockwood’s FI and IMWG-FI.

	AIC	BIC	C-Index
Model A	83.45	89.78	0.7745
Model B	88.35	94.68	0.7493

Model A: multivariate Cox model for survival time, including Rockwood’s FI and adjusting for sex, ISS stage, and CIRS comorbidity index. Model B: multivariate Cox model for survival time, including IMWG-FI and adjusting for sex, ISS stage, and CIRS comorbidity index.

## Data Availability

The original data presented in the study are openly available in Zenodo at https://zenodo.org/records/14197400 (accessed on 20 November 2024).

## Supplementary Materials

The following supporting information can be downloaded at: https://www.mdpi.com/article/10.3390/cancers17050789/s1, File S1: Tools used in our CGA. Refs. [31,33,34,35,39,42,44,50,51,52,53,54] are cited in the Supplementary File S1; File S2: Laboratoristic and MM-specific characteristics of the population; File S3. Univariable Cox proportional hazards model for death.
