# Supplementary material for: Prognostic Frailty-Based Determinants of Long-Term Mortality in Older Patients with Newly Diagnosed Multiple Myeloma
Source: Cancers (Basel). 2025 Feb 25;17(5):789. doi: 10.3390/cancers17050789 (PMC11898973; doi:10.3390/cancers17050789)
Supplement: Supplementary file 1 [file cancers-17-00789-s001.zip › File S1.pdf]

SUPPLEMENTARY MATERIAL

S1 Comprehensive Geriatric Assessment (CGA)

S1.1 Mini Mental State Examination (MMSE)

Maximum	Score	
		**Orientation**
5	( )	What is the (year) (season) (date) (day) (month)?
5	( )	Where are we (state) (country) (town) (hospital) (floor)?
		**Registration**
3	( )	Name 3 objects: 1 second to say each. Then ask the patient all 3 after you have said them. Give 1 point for each correct answer. Then repeat them until he/she learns all 3. Count trials and record. Trials ___________
		**Attention and Calculation**
5	( )	Serial 7's. 1 point for each correct answer. Stop after 5 answers. Alternatively spell "world" backward.
		**Recall**
3	( )	Ask for the 3 objects repeated above. Give 1 point for each correct answer.
		**Language**
2	( )	Name a pencil and watch.
1	( )	Repeat the following "No ifs, ands, or buts"
3	( )	Follow a 3-stage command: "Take a paper in your hand, fold it in half, and put it on the floor."
1	( )	Read and obey the following: CLOSE YOUR EYES
1	( )	Write a sentence.
1	( )	Copy the design shown.

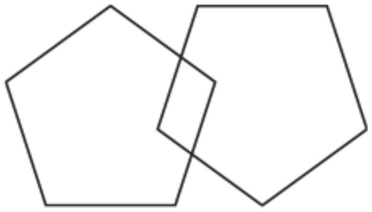

	_____	Total Score

Folstein MF, Folstein SE, McHugh PR. "Mini-mental state". A practical method for grading the cognitive state of patients for the clinician. J Psychiatr Res 1975; 12: 189-198.

S1.2 Barthel Index

THE BARTHEL INDEX

Patient Name: ____________________________

Rater Name: ____________________________

Date: ____________________________

Activity	Score
**FEEDING**<br>0 = unable<br>5 = needs help cutting, spreading butter, etc., or requires modified diet<br>10 = independent	______
**BATHING**<br>0 = dependent<br>5 = independent (or in shower)	______
**GROOMING**<br>0 = needs to help with personal care<br>5 = independent face/hair/teeth/shaving (implements provided)	______
**DRESSING**<br>0 = dependent<br>5 = needs help but can do about half unaided<br>10 = independent (including buttons, zips, laces, etc.)	______
**BOWELS**<br>0 = incontinent (or needs to be given enemas)<br>5 = occasional accident<br>10 = continent	______
**BLADDER**<br>0 = incontinent, or catheterized and unable to manage alone<br>5 = occasional accident<br>10 = continent	______
**TOILET USE**<br>0 = dependent<br>5 = needs some help, but can do something alone<br>10 = independent (on and off, dressing, wiping)	______
**TRANSFERS (BED TO CHAIR AND BACK)**<br>0 = unable, no sitting balance<br>5 = major help (one or two people, physical), can sit<br>10 = minor help (verbal or physical)<br>15 = independent	______
**MOBILITY (ON LEVEL SURFACES)**<br>0 = immobile or < 50 yards<br>5 = wheelchair independent, including corners, > 50 yards<br>10 = walks with help of one person (verbal or physical) > 50 yards<br>15 = independent (but may use any aid; for example, stick) > 50 yards	______
**STAIRS**<br>0 = unable<br>5 = needs help (verbal, physical, carrying aid)<br>10 = independent	______

TOTAL (0–100): _____

Mahoney FI, Barthel DW. Functional Evaluation: The Barthel Index. Md State Med J. 1965 Feb;14:61-5. PMID: 14258950.

S1.3 Instrumental Activities of Daily Living (IADL)

LAWTON - BRODY<br>INSTRUMENTAL ACTIVITIES OF DAILY LIVING SCALE (I.A.D.L.)			
**Scoring:** For each category, circle the item description that most closely resembles the client's highest functional level (either 0 or 1).			
**A. Ability to Use Telephone**		**E. Laundry**	
1. Operates telephone on own initiative-looks up and dials numbers, etc.<br>2. Dials a few well-known numbers<br>3. Answers telephone but does not dial<br>4. Does not use telephone at all	1<br>1<br>1<br>0	1. Does personal laundry completely<br>2. Launders small items-rinses stockings, etc.<br>3. All laundry must be done by others	1<br>1<br>0
**B. Shopping**		**F. Mode of Transportation**	
1. Takes care of all shopping needs independently<br>2. Shops independently for small purchases<br>3. Needs to be accompanied on any shopping trip<br>4. Completely unable to shop	1<br>0<br>0<br>0	1. Travels independently on public transportation or drives own car<br>2. Arranges own travel via taxi, but does not otherwise use public transportation<br>3. Travels on public transportation when accompanied by another<br>4. Travel limited to taxi or automobile with assistance of another<br>5. Does not travel at all	1<br>1<br>1<br>0<br>0
**C. Food Preparation**		**G. Responsibility for Own Medications**	
1. Plans, prepares and serves adequate meals independently<br>2. Prepares adequate meals if supplied with ingredients<br>3. Heats, serves and prepares meals, or prepares meals, or prepares meals but does not maintain adequate diet<br>4. Needs to have meals prepared and served	1<br>0<br>0<br>0	1. Is responsible for taking medication in correct dosages at correct time<br>2. Takes responsibility if medication is prepared in advance in separate dosage<br>3. Is not capable of dispensing own medication	1<br>0<br>0
**D. Housekeeping**		**H. Ability to Handle Finances**	
1. Maintains house alone or with occasional assistance (e.g. "heavy work domestic help")<br>2. Performs light daily tasks such as dish washing, bed making<br>3. Performs light daily tasks but cannot maintain acceptable level of cleanliness<br>4. Needs help with all home maintenance tasks<br>5. Does not participate in any housekeeping tasks	1<br>1<br>1<br>1<br>0	1. Manages financial matters independently (budgets, writes checks, pays rent, bills, goes to bank), collects and keeps track of income<br>2. Manages day-to-day purchases, but needs help with banking, major purchases, etc.<br>3. Incapable of handling money	1<br>1<br>0

Katz S. Assessing self-maintenance: activities of daily living, mobility, and instrumental activities of daily living. J Am Geriatr Soc. 1983 Dec;31(12):721-7. doi: 10.1111/j.1532-415.1983.tb03391.x

S1.4 Mini Nutritional Assessment (MNA)

Mini Nutritional Assessment MNA®

Nestlé NutritionInstitute

Last name: ____ First name: ____

Sex: ____ Age: ____ Weight, kg: ____ Height, cm: ____ Date: ____

Complete the screen by filling in the boxes with the appropriate numbers.
Add the numbers for the screen. If score is 11 or less, continue with the assessment to gain a Malnutrition Indicator Score.

Screening

A Has food intake declined over the past 3 months due to loss of appetite, digestive problems, chewing or swallowing difficulties?
0 = severe decrease in food intake
1 = moderate decrease in food intake
2 = no decrease in food intake ☐

B Weight loss during the last 3 months
0 = weight loss greater than 3kg (6.6lbs)
1 = does not know
2 = weight loss between 1 and 3kg (2.2 and 6.6 lbs)
3 = no weight loss ☐

C Mobility
0 = bed or chair bound
1 = able to get out of bed / chair but does not go out
2 = goes out ☐

D Has suffered psychological stress or acute disease in the past 3 months?
0 = yes 2 = no ☐

E Neuropsychological problems
0 = severe dementia or depression
1 = mild dementia
2 = no psychological problems ☐

F Body Mass Index (BMI) = weight in kg / (height in m)^2 ☐
0 = BMI less than 19
1 = BMI 19 to less than 21
2 = BMI 21 to less than 23
3 = BMI 23 or greater ☐

Screening score (subtotal max. 14 points) ☐☐
12-14 points: ☐ Normal nutritional status
8-11 points: ☐ At risk of malnutrition
0-7 points: ☐ Malnourished
For a more in-depth assessment, continue with questions G-R

Assessment

G Lives independently (not in nursing home or hospital)
1 = yes 0 = no ☐

H Takes more than 3 prescription drugs per day
0 = yes 1 = no ☐

I Pressure sores or skin ulcers
0 = yes 1 = no ☐

J How many full meals does the patient eat daily?
0 = 1 meal
1 = 2 meals
2 = 3 meals ☐

K Selected consumption markers for protein intake
- At least one serving of dairy products (milk, cheese, yoghurt) per day — yes ☐ no ☐
- Two or more servings of legumes or eggs per week — yes ☐ no ☐
- Meat, fish or poultry every day — yes ☐ no ☐

0.0 = if 0 or 1 yes
0.5 = if 2 yes
1.0 = if 3 yes ☐.☐

L Consumes two or more servings of fruit or vegetables per day?
0 = no 1 = yes ☐

M How much fluid (water, juice, coffee, tea, milk...) is consumed per day?
0.0 = less than 3 cups
0.5 = 3 to 5 cups
1.0 = more than 5 cups ☐.☐

N Mode of feeding
0 = unable to eat without assistance
1 = self-fed with some difficulty
2 = self-fed without any problem ☐

O Self view of nutritional status
0 = views self as being malnourished
1 = is uncertain of nutritional state
2 = views self as having no nutritional problem ☐

P In comparison with other people of the same age, how does the patient consider his / her health status?
0.0 = not as good
0.5 = does not know
1.0 = as good
2.0 = better ☐.☐

Q Mid-arm circumference (MAC) in cm
0.0 = MAC less than 21
0.5 = MAC 21 to 22
1.0 = MAC greater than 22 ☐.☐

R Calf circumference (CC) in cm
0 = CC less than 31
1 = CC 31 or greater ☐

Assessment (max. 16 points) ☐☐.☐
Screening score ☐☐.☐
Total Assessment (max. 30 points) ☐☐.☐

Malnutrition Indicator Score

24 to 30 points	☐	Normal nutritional status
17 to 23.5 points	☐	At risk of malnutrition
Less than 17 points	☐	Malnourished

References
1. Vellas B, Villars H, Abellan G, *et al.* Overview of the MNA® - Its History and Challenges. *J Nutr Health Aging.* 2006; **10:**456-465.
2. Rubenstein LZ, Harker JO, Salva A, Guigoz Y, Vellas B. Screening for Undernutrition in Geriatric Practice: Developing the Short-Form Mini Nutritional Assessment (MNA-SF). *J. Geront.* 2001; **56A**: M366-377
3. Guigoz Y. The Mini-Nutritional Assessment (MNA®) Review of the Literature - What does it tell us? *J Nutr Health Aging.* 2006; **10**:466-487.

® Société des Produits Nestlé SA, Trademark Owners
© Société des Produits Nestlé SA 1994, Revision 2009.

For more information: www.mna-elderly.com

Save Print Reset

Rubenstein LZ, Harker JO, Salvà A, Guigoz Y, Vellas B. Screening for undernutrition in geriatric practice: developing the short-form mini-nutritional assessment (MNA-SF). J Gerontol A Biol Sci Med Sci. 2001 Jun;56(6):M366-72. doi: 10.1093/gerona/56.6.m366.

S1.5 Cumulative Illness Rating Scale (CIRS)

Each system is rated as follows:

1 = NONE:	No impairment to that organ/system.
2 = MILD:	Impairment does not interfere with normal activity; treatment may not be required; prognosis is excellent (examples: skin lesions, hernias, hemorrhoids)
3 = MODERATE:	Impairment interferes with normal activity; treatment is needed; prognosis is good (examples: gallstones, diabetes, fractures)
4 = SEVERE:	Impairment is disabling; treatment is urgently needed; prognosis is guarded (examples: respectable carcinoma, pulmonary emphysema, congestive heart failure)
5 = EXTREMELY SEVERE:	Impairment is life threatening; treatment is urgent or of no avail; prognosis is grave (examples: myocardial infarction, cerebrovascular accident, gastrointestinal bleeding, embolus)

		Value 1-5
a.	Cardiac (heart only)	___
b.	Hypertension (rating is based on severity; affected systems are rated separately).	___
c.	Vascular (blood, blood vessels and cells, marrow, spleen, lymphatics).	___
d.	Respiratory (lungs, bronchi, trachea below the larynx).	___
e.	EENT (eye, ear, nose, throat, larynx).	___
f.	Upper GI (esophagus, stomach, duodenum, biliary and pancreatic trees; do no include diabetes).	___
g.	Lower GI (intestines, hernias).	___
h.	Hepatic (liver only).	___
i.	Renal (kidneys only).	___
j.	Other GU (ureters, bladder, urethra, prostate, genitals).	___
k.	Musculo-skeletal-integumentary (muscles, bone, skin)	___
l.	Neurological (brain, spinal cord, nerves; do not include dementia).	___
m.	Endocrine-Metabolic (includes diabetes, diffuse infections, infections, toxicity)	___
n.	Psychiatric/Behavioral (includes depression, anxiety, agitation, psychosis, not dementia).	___

D:\english\forms\cirs.doc

Linn BS, Linn MW, Gurel L. Cumulative illness rating scale. J Am Geriatr Soc. 1968 May;16(5):622-6. doi: 10.1111/j.1532-5415.1968.tb02103.x.

S1.6 Geriatric Depression Scale (Short Form) (GDS)

**1.**	Are you basically satisfied with your life?	yes	no
**2.**	Have you dropped many of your activities and interests?	yes	no
**3.**	Do you feel that your life is empty?	yes	no
**4.**	Do you often get bored?	yes	no
**5.**	Are you in good spirits most of the time?	yes	no
**6.**	Are you afraid that something bad is going to happen to you?	yes	no
**7.**	Do you feel happy most of the time?	yes	no
**8.**	Do you often feel helpless?	yes	no
**9.**	Do you prefer to stay at home, rather than going out and doing things?	yes	no
**10.**	Do you feel that you have more problems with memory than most?	yes	no
**11.**	Do you think it is wonderful to be alive now?	yes	no
**12.**	Do you feel worthless the way you are now?	yes	no
**13.**	Do you feel full of energy?	yes	no
**14.**	Do you feel that your situation is hopeless?	yes	no
**15.**	Do you think that most people are better off than you are?	yes	no

Yesavage JA, Sheikh JI. 9/Geriatric Depression Scale (GDS). Clinical Gerontologist 1986; 5: 165-173.

S1.7 Frailty Index according to Rockwood 40-items tool (FI -40 item)

ROCKWOOD FRAILTY INDEX

1. Help bathing				YES=1	NO=0
2. Help dressing				YES=1	NO=0
3. Help getting in/out of Chair				YES=1	NO=0
4. Help Walking around house				YES=1	NO=0
5. Help eating				YES=1	NO=0
6. Help Grooming				YES=1	NO=0
7. Help Using Toilet				YES=1	NO=0
8. Help up/down Stairs				YES=1	NO=0
9. Help lifting 10 lbs				YES=1	NO=0
10. Help Shopping				YES=1	NO=0
11. Help with Housework				YES=1	NO=0
12. Help with meal Preparations				YES=1	NO=0
13. Help taking Medication				YES=1	NO=0
14. Help with Finances				YES=1	NO=0
15. Lost more than 10 lbs in last year				YES=1	NO=0
16. Self Rating of Health	Poor = 1	Fair = 0.75	Good = 0.5	V. Good = 0.25	Excellent = 0
17. How Health has changed in last year				Worse = 1	Better/Same = 0
18. Stayed in Bed at least half the day due to health (in last month)				YES=1	NO=0
19. Cut down on Usual Activity (in last month)				YES=1	NO=0
20. Walk outside				<3 days =1	≥3 days = 0
21. Feel Everything is an Effort			Most of time = 1	Some time = 0.5	Rarely = 0
22. Feel Depressed			Most of time = 1	Some time = 0.5	Rarely = 0
23. Feel Happy			Most of time = 0	Some time = 0.5	Rarely = 1
24. Feel Lonely			Most of time = 1	Some time = 0.5	Rarely = 0
25. Have Trouble getting going			Most of time = 1	Some time = 0.5	Rarely = 0
26. High blood pressure			Yes = 1	Suspect = 0.5	No=0
27. Heart attack			Yes = 1	Suspect = 0.5	No=0

28. Cardiac heart failure			Yes = 1	Suspect = 0.5	No=0
29. Stroke			Yes = 1	Suspect = 0.5	No=0
30. Cancer			Yes = 1	Suspect = 0.5	No=0
31. Diabetes			Yes = 1	Suspect = 0.5	No=0
32. Arthritis			Yes = 1	Suspect = 0.5	No=0
33. Chronic Lung Disease			Yes = 1	Suspect = 0.5	No=0
34. MMSE	≤10=1	11-17= 0.75	18-20=0.5	21-24= 0.25	≥25=0
35. Peak Flow (liters/min)				Deficit for men=1 ≤ 340	Deficit for Women=1 ≤ 310
36. Shoulder Strength (kg)				Deficit for men=1 ≤ 12	Deficit for women=1 ≤ 9
37. Body Mass Index (BMI)				Deficit for men=1 <18.5, ≥ 30 as a deficit. 25-<30 as a 'half deficit'	Deficit for women <18.5, ≥ 30 as a deficit. 25-<30 as a 'half deficit'
38. Grip Strength (GS in kg)				Deficit for men=1<br>For BMI ≤ 24, GS ≤ 29<br>For BMI 24.1–28, GS ≤ 30<br>For BMI >28, GS ≤ 32	Deficit for women=1<br>For BMI ≤ 23, GS ≤ 17<br>For BMI 23.1–26, GS ≤ 17.3<br>For BMI 26.1–29, GS ≤ 18<br>For BMI>29, GS ≤ 21
39. Usual pace Walk (sec)				Deficit for men=1 >16	Deficit for women=1 >16
40. Rapid pace Walk (sec)				Deficit for men=1 >10	Deficit for women=1 >10

→ ..../40 → ..../1

NOT FRAIL ≤ 0,08
0,08 > **PREFRAIL** < 0,25
FRAIL ≥ 0,25

Searle SD, Mitnitski A, Gahbauer EA, Gill TM, Rockwood K. A standard procedure for creating a frailty index. BMC Geriatr. 2008 Sep 30;8:24. doi: 10.1186/1471-2318-8-24.

S1.8 Charlson Comorbidity Index

Comorbidity	Score
Prior myocardial infarction	1
Congestive heart failure	1
Peripheral vascular disease	1
Cerebrovascular disease	1
Dementia	1
Chronic pulmonary disease	1
Rheumatologic disease	1
Peptic ulcer disease	1
Mild liver disease	1
Diabetes	1
Cerebrovascular (hemiplegia) event	2
Moderate-to-severe renal disease	2
Diabetes with chronic complications	2
Cancer without metastases	2
Leukemia	2
Lymphoma	2
Moderate or severe liver disease	3
Metastatic solid tumor	6
Acquired immuno-deficiency syndrome (AIDS)	6

Charlson ME, Pompei P, Ales KL, MacKenzie CR. A new method of classifying prognostic comorbidity in longitudinal studies: development and validation. J Chronic Dis. 1987;40(5):373-83. doi: 10.1016/0021-9681(87)90171-8. PMID: 3558716.

S1.9 Activities of Daily Living (ADL)

Katz Index of Independence in Activities of Daily Living

**Activities**<br>Points (1 or 0)	**Independence**<br>(1 Point)<br><br>**NO** supervision, direction or personal assistance.	**Dependence**<br>(0 Points)<br><br>**WITH** supervision, direction, personal assistance or total care.
**BATHING**<br><br>Points: __________	**(1 POINT)** Bathes self completely or needs help in bathing only a single part of the body such as the back, genital area or disabled extremity.	**(0 POINTS)** Need help with bathing more than one part of the body, getting in or out of the tub or shower. Requires total bathing
**DRESSING**<br><br>Points: __________	**(1 POINT)** Get clothes from closets and drawers and puts on clothes and outer garments complete with fasteners. May have help tying shoes.	**(0 POINTS)** Needs help with dressing self or needs to be completely dressed.
**TOILETING**<br><br>Points: __________	**(1 POINT)** Goes to toilet, gets on and off, arranges clothes, cleans genital area without help.	**(0 POINTS)** Needs help transferring to the toilet, cleaning self or uses bedpan or commode.
**TRANSFERRING**<br><br>Points: __________	**(1 POINT)** Moves in and out of bed or chair unassisted. Mechanical transfer aids are acceptable	**(0 POINTS)** Needs help in moving from bed to chair or requires a complete transfer.
**CONTINENCE**<br><br>Points: __________	**(1 POINT)** Exercises complete self control over urination and defecation.	**(0 POINTS)** Is partially or totally incontinent of bowel or bladder
**FEEDING**<br><br>Points: __________	**(1 POINT)** Gets food from plate into mouth without help. Preparation of food may be done by another person.	**(0 POINTS)** Needs partial or total help with feeding or requires parenteral feeding.

TOTAL POINTS: ________ **SCORING:** 6 = High (*patient independent*) 0 = Low (*patient very dependent*

Katz, S. (1983). Assessing self-maintenance: Activities of daily living, mobility and instrumental activities of daily living. JAGS, 31(12), 721-726.

S1.10 EuroQoL

Health Questionnaire (EQ-5D-5L)

Under each heading, please tick the ONE box that best describes your health TODAY.

MOBILITY

- ☐ 1 I have no problems in walking about
- ☐ 2 I have slight problems in walking about
- ☐ 3 I have moderate problems in walking about
- ☐ 4 I have severe problems in walking about
- ☐ 5 I am unable to walk about

SELF-CARE

- ☐ 1 I have no problems washing or dressing myself
- ☐ 2 I have slight problems washing or dressing myself
- ☐ 3 I have moderate problems washing or dressing myself
- ☐ 4 I have severe problems washing or dressing myself
- ☐ 5 I am unable to wash or dress myself

USUAL ACTIVITIES *(e.g. work, study, housework, family or leisure activities)*

- ☐ 1 I have no problems doing my usual activities
- ☐ 2 I have slight problems doing my usual activities
- ☐ 3 I have moderate problems doing my usual activities
- ☐ 4 I have severe problems doing my usual activities
- ☐ 5 I am unable to do my usual activities

PAIN / DISCOMFORT

- ☐ 1 I have no pain or discomfort
- ☐ 2 I have slight pain or discomfort
- ☐ 3 I have moderate pain or discomfort
- ☐ 4 I have severe pain or discomfort
- ☐ 5 I have extreme pain or discomfort

ANXIETY / DEPRESSION

- ☐ 1 I am not anxious or depressed
- ☐ 2 I am slightly anxious or depressed
- ☐ 3 I am moderately anxious or depressed
- ☐ 4 I am severely anxious or depressed
- ☐ 5 I am extremely anxious or depressed

UK (English) © 2009 EuroQol Group EQ-5D™ is a trade mark of the EuroQol Group

EuroQol Group (1990) EuroQol--a new facility for the measurement of health-related quality of life. *Health Policy Amst Neth* **16**, 199–208.

S1.11 Tinetti Balance and Gait Assessment Tool

TINETTI BALANCE ASSESSMENT TOOL

Tinetti ME, Williams TF, Mayewski R, Fall Risk Index for elderly patients based on number of chronic disabilities. Am J Med 1986:80:429-434

PATIENTS NAME ____________________ D.o.b. __________ Ward ______

BALANCE SECTION

Patient is seated in hard, armless chair;

	**Date**		
Sitting Balance	Leans or slides in chair = 0<br>Steady, safe = 1		
Rises from chair	Unable to without help = 0<br>Able, uses arms to help = 1<br>Able without use of arms = 2		
Attempts to rise	Unable to without help = 0<br>Able, requires > 1 attempt = 1<br>Able to rise, 1 attempt = 2		
Immediate standing Balance (first 5 seconds)	Unsteady (staggers, moves feet, trunk sway) = 0<br>Steady but uses walker or other support = 1<br>Steady without walker or other support = 2		
Standing balance	Unsteady = 0<br>Steady but wide stance and uses support = 1<br>Narrow stance without support = 2		
Nudged	Begins to fall = 0<br>Staggers, grabs, catches self = 1<br>Steady = 2		
Eyes closed	Unsteady = 0<br>Steady = 1		
Turning 360 degrees	Discontinuous steps = 0<br>Continuous = 1		
	Unsteady (grabs, staggers) = 0<br>Steady = 1		
Sitting down	Unsafe (misjudged distance, falls into chair) = 0<br>Uses arms or not a smooth motion = 1<br>Safe, smooth motion = 2		
	**Balance score**	/16	/16

[illegible]

TINETTI BALANCE ASSESSMENT TOOL

GAIT SECTION

Patient stands with therapist, walks across room (+/- aids), first at usual pace, then at rapid pace.

	**Date**		
Indication of gait (Immediately after told to 'go'.)	Any hesitancy or multiple attempts = 0<br>No hesitancy = 1		
Step length and height	Step to = 0<br>Step through R = 1<br>Step through L = 1		
Foot clearance	Foot drop = 0<br>L foot clears floor = 1<br>R foot clears floor = 1		
Step symmetry	Right and left step length not equal = 0<br>Right and left step length appear equal = 1		
Step continuity	Stopping or discontinuity between steps = 0<br>Steps appear continuous = 1		
Path	Marked deviation = 0<br>Mild/moderate deviation or uses w. aid = 1<br>Straight without w. aid = 2		
Trunk	Marked sway or uses w. aid = 0<br>No sway but flex. knees or back or uses arms for stability = 1<br>No sway, flex., use of arms or w. aid = 2		
Walking time	Heels apart = 0<br>Heels almost touching while walking = 1		
	**Gait score**	/12	/12
	**Balance score carried forward**	/16	/16
	**Total Score = Balance + Gait score**	/28	/28

Risk Indicators:

**Tinetti Tool Score**	**Risk of Falls**
≤18	High
19-23	Moderate
≥24	Low

51

Tinetti ME (1986) Performance-oriented assessment of mobility problems in elderly patients. *J Am Geriatr Soc* **34**, 119–126.

S1.12 Timed Up and Go Test (TUG)

Timed Up and Go (TUG) Test[1,2]

1. Equipment: arm chair, tape measure, tape, stop watch.

2. Begin the test with the subject sitting correctly in a chair with arms, the subject's back should resting on the back of the chair. The chair should be stableand positioned such that it will not move when the subject moves from sitting to standing.

3. Place a piece of tape or other marker on the floor 3 meters away from the chair so that it is easily seen by the subject.

4. Instructions : "On the word *GO* you will stand up, walk to the line on the floor, turn around and walk back to the chair and sit down. Walk at your regular pace.

5. Start timing on the word "*GO*" and stop timing when the subject is seated again correctly in the chair with their back resting on the back of the chair.

6. The subject wears their regular footwear, may use any gait aid that they normally use during ambulation, but may not be assisted by another person. There is no time limit. They may stop and rest (but not sit down) if they need to.

7. Normal healthy elderly usually complete the task in ten seconds or less. Very frail or weak elderly with poor mobility may take 2 minutes or more.

8. The subject should be given a practice trial that is not timed before testing.

9. Results correlate with gait speed, balance, functional level,the ability to go out, and can follow change over time.

10. Interpretation ≤ 10 seconds = normal

≤ 20 seconds = good mobility, can go out alone, mobile without a gait aid.

< 30 seconds = problems, cannot go outside alone, requires a gait aid.

A score of more than or equal to fourteen seconds has been shown to indicate high risk of falls.

1. Podsiadlo D, Richardson S. *The Time "Up & Go": A Test of Basic Functional Mobility for Frail Elderly Persons.* Journal of the American Geriatrics Society 1991; 39(2): 142-148
2. Shumway - Cook A, Brauer S, Woollacott M. *Predicting the Probability for Falls in Community-Dwelling Older Adults Using the Timed Up & Go Test. Physical Therapy 2000 Vol 80(9): 896-903.*
Saskatoon Falls Prevention Consortium, Falls Screening and Referral Algorithm, TUG, Saskatoon Falls Prevention consortium, June, 2005

S1.12 G8 questionnaire

G8 questionnaire

	Items	Possible answers (score)
**A**	Has food intake declined over the past 3 months due to loss of appetite, digestive problems, chewing or swallowing difficulties?	0 : severe decrease in food intake
		1 : moderate decrease in food intake
		2 : no decrease in food intake
**B**	Weight loss during the last 3 months	0 : weight loss > 3 kg
		1 : does not know
		2 : weight loss between 1 and 3 kgs
		3 : no weight loss
**C**	Mobility	0 : bed or chair bound
		1 : able to get out of bed/chair but does not go out
		2 : goes out
**E**	Neuropsychological problems	0 : severe dementia or depression
		1 : mild dementia or depression
		2 : no psychological problems
**F**	Body Mass Index (BMI (weight in kg) / (height in m^2)	0 : BMI < 19
		1 : BMI = 19 to BMI < 21
		2 : BMI = 21 to BMI < 23
		3 : BMI = 23 and > 23
**H**	Takes more than 3 medications per day	0 : yes
		1 : no
**P**	In comparison with other people of the same age, how does the patient consider his/her health status?	0 : not as good
		0.5 : does not know
		1 : as good
		2 : better
	Age	0 : >85
		1 : 80-85
		2 : <80
	**TOTAL SCORE**	**0 – 17**
