# Supplementary material for: Prognostic Frailty-Based Determinants of Long-Term Mortality in Older Patients with Newly Diagnosed Multiple Myeloma
Source: Cancers (Basel). 2025 Feb 25;17(5):789. doi: 10.3390/cancers17050789 (PMC11898973; doi:10.3390/cancers17050789)
Supplement: Supplementary file 1 [file cancers-17-00789-s001.zip › File S2_Table.docx]

**Laboratoristic and MM-specific characteristics of the population.**

|  | **Overall,**  **N=36 (100%)** | **Dead,**  **N=14 (39%)** | **Alive,**  **N=22 (61%)** | **p-value** |
| --- | --- | --- | --- | --- |
| White blood cells, median [IQR] | N=35  5.9 [4.52-7.49] | N=14  6.22 [4.52-9.09] | N=21  5.9 [4.72-6.52] | 0.363 |
| Hb | 10.94 (1.81) | 11.49 (1.94) | 10.60 (1.67) | 0.153 |
| PLT | N=35  200.28 (85.82) | N=14  224.79 (82.95) | N=21  183.94 (85.72) | 0.171 |
| Creatinine, median [IQR] | 1.2 [1-1.6] | 1.3 [1-1.5] | 1.2 [1-1.8] | 0.948 |
| Urea, median [IQR] | N=31  48 [33-72] | N=14  51 [36-78] | N=17  47 [33-57] | 0.487 |
| Urine protein g/24h, median [IQR] | N=29  0.69 [0.24-1.85] | N=11  0.64 [0.34-1.37] | N=18  0.72 [0.23-2.31] | 0.840 |
| INR, median [IQR] | N=22  1.07 [1.02-1.11] | N=7  1.06 [1.02-1.33] | N=15  1.07 [1.02-1.11] | 0.671 |
| PT, median [IQR] | N=17  91 [76-97] | N=7  91 [26-97] | N=10  89.5 [80-99] | 0.406 |
| PTT, median [IQR] | N=21  30.3 [28.1-31.6] | N=7  30.8 [28.7-32.7] | N=14  29.1 [27.8-31.4] | 0.247 |
| Calcium, median [IQR] | N=33  9.5 [9.2-9.9] | N=13  9.8 [9.5-10.1] | N=20  9.4 [9.05-9.6] | **0.040** |
| Albumin | N=35  37.37 (5.49) | N=14  35.89 (6.45) | N=21  38.36 (4.65) | 0.197 |
| Total proteins | N=33  86.36 (15.69) | N=13  88.22 (21.11) | N=20  85.15 (11.37) | 0.592 |
| Total bilirubin | N=30  0.50 (0.24) | N=13  0.61 (0.29) | N=17  0.41 (0.17) | **0.023** |
| Direct bilirubin, median [IQR] | N=19  0.2 [0.1-0.3] | N=9  0.2 [0.2-0.3] | N=10  0.1 [0.1-0.2] | **0.028** |
| ALT, median [IQR] | N=31  16 [11-22] | N=13  16 [11-19] | N=18  17 [11-23] | 0.749 |
| B2 mg/L, median [IQR] | N=34  6.05 [3.8-10.4] | N=14  7.25 [3.8-10.4] | N=20  5.75 [3.85-10.45] | 0.740 |
| LDH, median [IQR] | N=34  169.5 [151-215] | N=13  165 [136-223] | N=21  170 [155-205] | 0.763 |
| sFLC (mg/L), median [IQR] | N=35  313 [113-901] | N=14  492.5 [113-1210] | N=21  299 [150-567] | 0.590 |
| CM (g/L), median [IQR] | 27.4 [12.35-38.8] | 29.35 [11.1-45.4] | 27.4 [15.9-36.96] | 0.650 |
| BJ 24h, present | 27 (75.00%) | 10 (71.43%) | 17 (77.27%) | 0.693 |
| Istotype |  |  |  | 0.802 |
| IgA | 9 (25.00%) | 4 (28.57%) | 5 (22.73%) |  |
| IgG | 23 (63.89%) | 9 (64.29%) | 14 (63.64%) |  |
| Micromolecular | 4 (11.11%) | 1 (7.14%) | 3 (13.64%) |  |
| Cytogenetic risk | N=24 | N=7 | N=17 | 0.344 |
| 1 (standard) | 17 (70.83%) | 4 (57.14%) | 13 (76.47%) |  |
| 3 (high risk) | 7 (29.17%) | 3 (42.86%) | 4 (23.53%) |  |
| Bone disease, yes | N=35  28 (80.00%) | N=13  13 (100.00%) | N=22  15 (68.18%) | **0.023** |
| Etramedullary/extraosseus disease, yes | N=35  2 (5.71%) | N=14  1 (7.14%) | N=21  1 (4.76%) | 0.766 |
| % PC, median [IQR] | N=21  6 [2-18] | N=10  7.5 [3.6-16] | N=11  5 [1-18] | 0.778 |
| Lymphocytes CD3, median [IQR] | N=21  13 [8-22] | N=10  14 [7-22] | N=11  10 [8-19] | 0.621 |
| Mature B lymphocyte, median [IQR] | N=21  1 [1-2] | N=10  1.4 [1-3] | N=11  1 [0.72-2] | 0.494 |
| Lymphocytes NK %, median [IQR] | N=21  2 [1-4] | N=10  1.6 [1-3.8] | N=11  2 [1-4] | 0.542 |
| Lymphocytes CD4, median [IQR] | N=21  7 [4-9] | N=10  7.05 [3.4-11] | N=11  7 [4-9] | 0.916 |
| Lymphocytes CD8, median [IQR] | N=21  4 [3-9] | N=10  8 [3-9] | N=11  4 [3-10] | 0.498 |
| CD4/CD8, median [IQR] | N=21  1.36 [0.8-1.94] | N=10 | N=11 | 0.339 |
| Monocytes | N=21  3.52 (2.09) | N=10  4.2 (2.20) | N=11  2.9 (1.87) | 0.162 |
| CD38 | N=21 | N=10 | N=11 | 0.329 |
| 2 (medium expression) | 1 (4.76%) | 0 (0.00%) | 1 (9.09%) |  |
| 3 (bright expression) | 20 (95.24%) | 10 (100%) | 10 (90.91%) |  |
| CD138 | N=21 | N=10 | N=11 | 0.537 |
| 2 (medium expression) | 14 (66.67%) | 6 (60.00%) | 8 (72.73%) |  |
| 3 (bright expression) | 7 (33.33%) | 4 (40.00%) | 3 (27.27%) |  |
| CD56 | N=21 | N=10 | N=11 | 0.633 |
| 0 (negative) | 7 (33.33%) | 4 (40.00%) | 3 (27.27%) |  |
| 1 (partial expression) | 3 (14.29%) | 2 (20.00%) | 1 (9.09%) |  |
| 2 (medium expression) | 7 (33.33%) | 2 (20.00%) | 5 (45.45%) |  |
| 3 (bright expression) | 4 (19.05%) | 2 (20.00%) | 2 (18.18%) |  |
| CD45 | N=21 | N=10 | N=11 | 0.157 |
| 0 (negative) | 5 (23.81%) | 1 (10.00%) | 4 (36.36%) |  |
| 1 (partial expression) | 16 (76.19%) | 9 (90.00%) | 7 (63.64%) |  |
| CD19 | N=21 | N=10 | N=11 | 0.366 |
| 0 (negative) | 19 (90.48%) | 9 (90.00%) | 10 (90.91%) |  |
| 1 (partial expression) | 1 (4.76%) | 0 (0.00%) | 1 (9.09%) |  |
| 2 (medium expression) | 1 (4.76%) | 1 (10.00%) | 0 (0.00%) |  |
| CD20 | N=21 | N=10 | N=11 | 0.296 |
| 0 (negative) | 19 (90.48%) | 8 (80.00%) | 11 (100.00%) |  |
| 1 (partial expression) | 1 (4.76%) | 1 (10.00%) | 0 (0.00%) |  |
| 2 (medium expression) | 1 (4.76%) | 1 (10.00%) | 0 (0.00%) |  |
| Restriction light chains | N=21 | N=10 | N=11 | 0.696 |
| kappa | 16 (76.19%) | 8 (80.00%) | 8 (72.73%) |  |
| lambda | 5 (23.81%) | 2 (20.00%) | 3 (27.27%) |  |

(i) the entire population; (ii) dead patients; (iii) alive patients. Results are presented through mean and standard deviation (SD) and absolute frequency (N) and relative frequency (%), for continuous and categorical variables respectively, unless otherwise specified. P-values of the t-test, the Wilcoxon sum-of-ranks test, or the chi-squared test are presented to compare (ii) and (iii), according to the distribution.

Abbreviation list: IQR: interquartile range; Hb: hemoglobin; PLT: Platelets; INR: international normalized ratio; PT: prothrombin time; PTT: partial thromboplastin time; ALT: alanine transaminase; B2: beta2-microglobulin; LDH: lactate dehydrogenase; sFLC: serum free light chains; CM: monoclonal component; BJ 24h: Bence Jones proteinuria over 24 hours; PC: plasma cells.
