# Supplementary material for: Prognostic Frailty-Based Determinants of Long-Term Mortality in Older Patients with Newly Diagnosed Multiple Myeloma
Source: Cancers (Basel). 2025 Feb 25;17(5):789. doi: 10.3390/cancers17050789 (PMC11898973; doi:10.3390/cancers17050789)
Supplement: Supplementary file 1 [file cancers-17-00789-s001.zip › File S3_Table.docx]

**Univariable Cox proportional hazards model for death.**

|  | **Univariable** | |
| --- | --- | --- |
|  | **HR(95% CI)** | **p-value** |
| White blood cells (N=35) | 1.18 (0.99-1.41) | 0.066 |
| Hb | 1.14 (0.83-1.57) | 0.407 |
| PLT (N=35) | 1.00 (1.00-1.01) | 0.084 |
| Creatinine | 1.12 (0.87-1.44) | 0.377 |
| Urea (N=31) | 1.01 (0.99-1.02) | 0.315 |
| Urine protein g/24h (N=29) | 0.91 (0.56-1.48) | 0.715 |
| INR (0.1 increase) (N=22) | 1.11 (0.99-1.25) | 0.075 |
| PT (N=17) | 0.98 (0.95-1.00) | 0.053 |
| PTT (N=21) | 1.15 (0.96-1.39) | 0.137 |
| Calcium (N=33) | 1.59 (0.97-2.60) | 0.065 |
| Albumin (N=35)* | 0.90 (0.81-0.99) | 0.044 |
| Total proteins (N=33) | 1.03 (0.98-1.07) | 0.228 |
| Total bilirubin (0.1 increase) (N=30) | 1.18 (0.97-1.43) | 0.097 |
| Direct bilirubin (0.1 increase) (N=19) | 1.38 (0.89-2.15) | 0.152 |
| ALT (N=31) | 1.00 (0.95-1.06) | 0.983 |
| B2 mg/L (N=34) | 1.01 (0.98-1.03) | 0.658 |
| LDH (N=34) | 1.00 (1.00-1.01) | 0.511 |
| sFLC (mg/L) (100 increase) (N=35) | 1.00 (0.96-1.05) | 0.899 |
| CM (g/L) | 1.02 (0.99-1.06) | 0.164 |
| BJ 24h (present vs. absent) | 1.00 (0.31-3.21) | 1.000 |
| Istotype |  |  |
| IgG vs. IgA | 0.61 (0.17-2.10) | 0.428 |
| micromolecular vs. IgA | 0.32 (0.03-3.01) | 0.322 |
| Cytogenetic risk (high risk vs. standard risk) (N=24) | 12.16 (1.20-122.90) | **0.034**** |
| Extramedullary/extraosseous disease (yes vs. no) (N=35) | 0.79 (0.10-6.16) | 0.825 |
| % PC (N=21) | 1.03 (0.98-1.09) | 0.227 |
| Lymphocytes CD3 (N=21) | 1.00 (0.94-1.07) | 0.945 |
| Mature B Lymphocytes (N=21) | 0.99 (0.69-1.41) | 0.943 |
| Lymphocytes NK (N=21) | 0.89 (0.71-1.12) | 0.326 |
| Lymphocytes CD4 (N=21) | 0.97 (0.85-1.10) | 0.599 |
| Lymphocytes CD8 (N=21) | 1.09 (0.92-1.29) | 0.301 |
| CD4/CD8 (0.1 increase) (N=21) | 0.95 (0.86-1.04) | 0.255 |
| Monocytes (N=21) | 1.24 (0.88-1.75) | 0.225 |
| CD138 (bright vs. medium) (N=21) | 1.21 (0.34-4.33) | 0.769 |
| CD56 (positive vs. negative) (N=21) | 0.56 (0.15-2.06) | 0.382 |
| CD45 (positive vs. negative) (N=21) | 3.11 (0.39-25.02) | 0.286 |
| CD19 (positive vs. negative) (N=21) | 0.56 (0.06-4.86) | 0.598 |
| CD20 (positive vs. negative) (N=21) | 3.54 (0.70-17.83) | 0.126 |
| Restriction light chains (lambda vs. kappa) (N=21) | 1.07 (0.21-5.32) | 0.939 |

HR: hazard ratio; CI: confidence interval.

* Excluded from the multivariable model due to the high correlation with another variable included.

** Excluded from the multivariable model due to the low number of subjects in the categories.
